# Research on Energy Dissipation Mechanism of Cobweb-like Disk Resonator Gyroscope

**DOI:** 10.3390/mi15111380

**Published:** 2024-11-15

**Authors:** Huang Yi, Bo Fan, Feng Bu, Fang Chen, Xiao-Qing Luo

**Affiliations:** 1 School of Electrical Engineering, University of South China, Hengyang 421001, China; yi1838139567@163.com (H.Y.); xqluophys@gmail.com (X.-Q.L.); 2 School of Electronic and Information Engineering, Soochow University, Suzhou 215006, China; 3 School of Electronic and Information Engineering, Suzhou Vocational University, Suzhou 215104, China; 92010@jssvc.edu.cn; 4 State Key Laboratory of Transducer Technology, Shanghai Institute of Microsystem and Information Technology, Chinese Academy of Sciences, Shanghai 200050, China; 5 Hunan Province Key Laboratory for Ultra-Fast Micro/Nano Technology, Advanced Laser Manufacture, University of South China, Hengyang 421001, China

**Keywords:** energy dissipation, disk resonator gyroscope, air damping, thermoelastic damping

## Abstract

The micro disk resonator gyroscope is a micro-mechanical device with potential for navigation-grade applications, where the performance is significantly influenced by the quality factor, which is determined by various energy dissipation mechanisms within the micro resonant structure. To enhance the quality factor, these gyroscopes are typically enclosed in high-vacuum packaging. This paper investigates a wafer-level high-vacuum-packaged (<0.1 Pa) cobweb-like disk resonator gyroscope, presenting a systematic and comprehensive theoretical analysis of the energy dissipation mechanisms, including air damping, thermoelastic damping, anchor loss, and other factors. Air damping is analyzed using both a continuous fluid model and an energy transfer model. The analysis results are validated through quality factor testing on batch samples and temperature characteristic testing on individual samples. The theoretical results obtained using the energy transfer model closely match the experimental measurements, with a maximum error in the temperature coefficient of less than 2%. The findings indicate that air damping and thermoelastic damping are the predominant energy dissipation mechanisms in the cobweb-like disk resonant gyroscope under high-vacuum conditions. Consequently, optimizing the resonator to minimize thermoelastic and air damping is crucial for designing high-performance gyroscopes.

## 1. Introduction

MEMS vibratory gyroscopes are angular velocity sensors that operate based on the Coriolis effect and micromachining technology. Due to their distinctive advantages in terms of their compact size, low power consumption, and cost-effectiveness, these gyroscopes are widely used across various fields, including inertial navigation, consumer electronics, automotive safety systems, and military fields [1]. Recently, disk resonator gyroscopes have garnered significant research interest due to their superior symmetry, high sensitivity, stable scale factor, and potential self-compensation capabilities [2]. The quality factor (*Q*) of these gyroscopes is a critical performance parameter that directly influences essential metrics such as sensitivity, noise, and bias instability [3].

The quality factor (*Q*) quantitatively represents the various energy dissipation mechanisms within a gyroscope. The energy dissipation in a gyroscope can be influenced by multiple factors, including thermal conduction in the surrounding air, energy dissipation through the anchor points, surface effect loss, electronic loss, thermoelastic loss, and phonon diffusion loss (Akhiezer loss). As these attenuation phenomena are independent and may manifest partially or fully within the resonator, the contributions of these dissipation mechanisms to the overall energy loss are fundamentally independent of one another. Consequently, the quality factor (*Q*) can be mathematically expressed as [4].

(1) $$\frac{1}{Q}=\frac{1}{Q_{air}}+\frac{1}{Q_{TED}}+\frac{1}{Q_{Anchor}}+\frac{1}{Q_{other}}$$

where *Q_air_*, *Q_TED_*, and *Q_anchor_* are the quality factors of gas loss, thermoelastic loss, and anchor loss, respectively; *Q_other_* is the quality factor of other energy dissipation mechanisms, such as the surface loss, electronic loss, and Akhiezer loss. The overall *Q* value of the resonator is typically determined by the predominant energy dissipation mechanism or the mechanism contributing the lowest *Q* value.

Currently, research on energy dissipation mechanisms primarily focuses on thermoelastic damping and anchor loss [5,6,7]. In high-vacuum-packaged gyroscopes, air damping is typically neglected. However, given the small structural gaps in these devices, the impact of air damping on gyroscope energy dissipation remains significant, even under high-vacuum conditions.

This paper presents a comprehensive analysis of the energy dissipation mechanisms in a MEMS cobweb-like disk resonant gyroscope. Air damping is assessed using a modified continuum fluid model and an energy transfer model, while thermoelastic damping is examined through the Zener analytical model and finite element simulations. The anchor loss is estimated using the Perfectly Matched Layer (PML) method. Other loss mechanisms are estimated using analytical models. Finally, experimental verifications are conducted to validate the theoretical analyses.

## 2. Design and Operation

This study introduces a novel cobweb-like disk resonant gyroscope (CDRG), developed in reference to the international standard ring-shaped disk resonant gyroscope design, but with a regular polygonal frame substituting for the traditional arc-shaped ring structure [8]. While the CDRG design comprises exclusively linear structures without any arcs, its structural properties are essentially consistent with those of conventional ring-shaped disk resonant gyroscopes.

**Figure 1:**
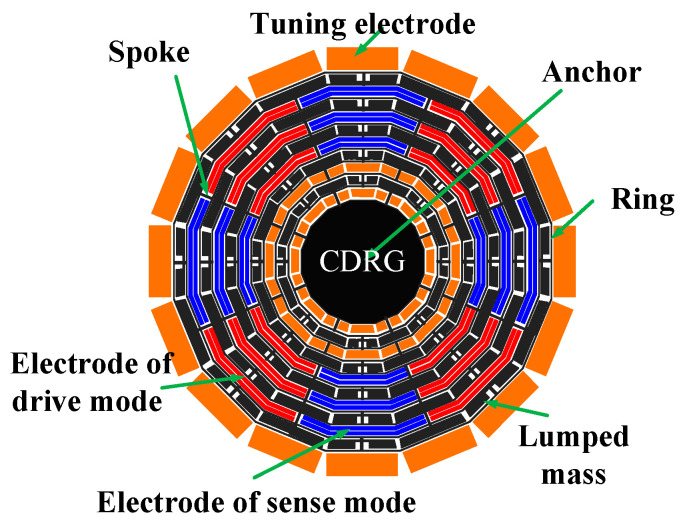
A cobweb-like disk resonator gyroscope (CDRG) structure schematic diagram with mass and stiffness decoupling.

The schematic diagram of the CDRG structure is illustrated in Figure 1. It comprises 10 concentric hexagonal rings, with the hexagon selected for convenience in the external electrode correspondence, though other regular polygon frames can be considered. These rings are alternately connected to the central anchor point through eight spokes. Each ring is connected end-to-end by 16 identical rectangular beams. Although the resonator appears as a disk with multiple rings, it is actually an all-linear structure. The diameter of the outermost ring measures 3.8 mm, while the diameter of the central anchor is 1.7 mm. A small solid mass, with a width of 77 μm, is suspended in both an odd-numbered ring and an even-numbered spoke layer, extending from the inside to the outside. The resonator’s thickness is 100 μm, and the widths of the spokes and rings are 13.5 μm and 13 μm, respectively. Surrounding the resonator are 16 uniformly distributed external electrodes for frequency tuning and orthogonal zeroing. There are eight independent slots between the rings, where internal double electrodes are inserted to enhance the conduction area for drive and detection. Additionally, a regulating electrode is inserted into the inner groove to improve the tuning capability, with a capacitance gap of 7.5 μm.

**Figure 2:**
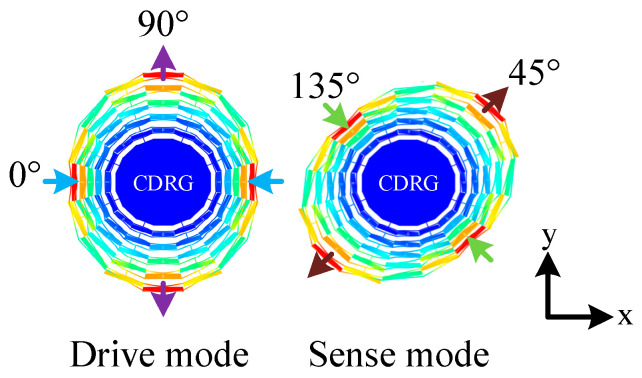
Mode shapes of the *n*  =  2 wineglass modes of CDRG.

The resonator is a planar structure, and the selected working vibration mode features a pair of in-plane elliptical bending. The wave-belly angle difference between the drive mode and the sense mode is 45°, while the phase difference is 90°. This vibration mode is commonly referred to as the wineglass mode, specifically, the *n* = 2 cup mode. The working mode shapes of the CDRG are illustrated in Figure 2, with the resonant frequency 18,188 Hz. The main structural parameters of the final design are summarized in Table 1.

## 3. Energy Dissipation Analysis

### 3.1. Air Damping

Due to the size effect, the volume of MEMS resonators decreases while the surface area-to-volume ratio increases, leading to a significant influence of the damping force on the surface. Many literature sources neglect the air damping loss mechanism in MEMS disk resonant gyroscopes, primarily due to vacuum packaging. However, even in an extremely low-pressure environment, the reduction in the resonator characteristic size leads to energy dissipation from interactions between air molecules and the resonator, making the air damping effect significant and worthy of attention [9].

Air damping can be classified into squeeze film damping and slide film damping, depending on whether the movement direction of the resonator is vertical or parallel to its surface. The primary mechanism of air damping for axially symmetric gyroscopes, which is the focus of this paper, is squeeze film damping. The degree of gas rarefaction affects the accuracy of the analytical model for squeeze film damping, necessitating a description of the gas rarefaction degree. Knudsen proposed the concept of the Knudsen number (*K_n_*) to characterize this degree, expressed as

(2) $$K_{n}=\lambda /d$$

where *d* is the characteristic size of the device and *λ* is the average free path of the gas molecules. According to the definition of *λ* and the ideal gas state equation, *K_n_* can be expressed as

(3) $$K_{n}=\frac{\lambda }{d}=\frac{BT}{pd}$$

Among them, $B=R/\sqrt{2}\pi d_{m}^{2}N_{A}$ is a constant. For air, the value of *B* is 2.2248 × 10^−5^ kg/(K·s^2^), *R* is the general gas constant (8.31 kg·m^2^/s^2^/K), N_A_ is the Avogadro constant, *d_m_* is the effective diameter of the gas molecules, *T* is the absolute temperature, and *p* is the gas pressure.

From Formula (3), it is evident that the Knudsen number is related to both the structural feature size and the ambient pressure. Based on the range of the Knudsen number, the flowing gas can be divided into four regions [10]: *K_n_* < 10^−3^ (continuous medium flow region), 10^−3^ < *K_n_* < 10^−1^ (slip flow region), 10^−1^ < *K_n_* < 10 (transition flow region), and *K_n_* > 10 (free molecular flow region).

Flow air damping arises from the viscosity of the fluid itself, represented by the viscosity coefficient. The Sutherland equation, an empirical formula proposed by W. Sutherland, describes the relationship between the viscosity coefficient *μ* and temperature [11].

(4) $$\mu =\mu_{0}\frac{1+T_{S}/T_{0}}{1+T_{S}/T}\sqrt{\frac{T}{T_{0}}}$$

Here, *T*_0_ = 237.16 K, *μ*_0_ represents the viscosity coefficient at *T*_0_, and *T_S_* is the constant temperature. The values of *μ*_0_ and *T_S_* depend on the specific gas. For air, *μ*_0_ is 1.85 × 10^−5^ Pa·s and *T_S_* is 124 K.

When *K_n_* > 10^−3^, the effective viscosity coefficient *μ_eff_* is introduced to correct *μ* when the N-S equation is used to solve the air damping model in the discontinuous medium flow region. The following formula is the approximate correction formula proposed by Veijola. The correction is in the range of 0 < *K_n_* < 880, and the deviation from the derivation result based on the Boltzmann transport equation is within 5% [12].

(5) $$\mu_{eff}=\frac{\mu }{1+9.638K_{n}^{1.159}}$$

The CDRG is vacuum-packaged and operates in the free molecular flow region. For the squeeze film damping effect in rarefied gas, two types of models can be used for analysis. The first is the gradient model, with the modified continuous fluid model based on the Reynolds equation being a typical example. The second is the non-gradient model, which is often represented by energy transfer models. The following discussion addresses these two models separately.

#### 3.1.1. Modified Continuous Fluid Model

Since the constitutive equation of the continuous fluid model has been well-established and validated, the squeeze film damping model based on the Reynolds equation is worth considering even in the free molecular flow state. The behavior of the squeeze film is generally governed by the viscous and inertial effects of the fluid. However, for the extremely small geometries typical of MEMS devices, the inertial effects are usually negligible. Under these conditions, the Reynolds equation can be applied with the following assumptions [13]:

- Rigid plate;
- Small gap;
- Small displacement;
- Small pressure change.

The Reynolds equation used to analyze squeeze film damping is expressed as

(6) $$\frac{ph^{2}}{12\mu }\nabla^{2}\left(\frac{\Delta p}{p}\right)-\frac{\partial }{\partial t}\left(\frac{\Delta p}{p}\right)=\frac{\partial }{\partial t}\left(\frac{\Delta h}{h}\right)$$

where *p* is the ambient pressure, Δ*p* is the distribution of pressure variation, *h* is the gap between the plates, and Δ*h* is the variation in gap height. The Reynolds equation describes the relationship between the pressure distribution and changes in film thickness. By applying specific boundary conditions, the pressure at each point can be determined by solving this second-order differential equation.

**Figure 3:**
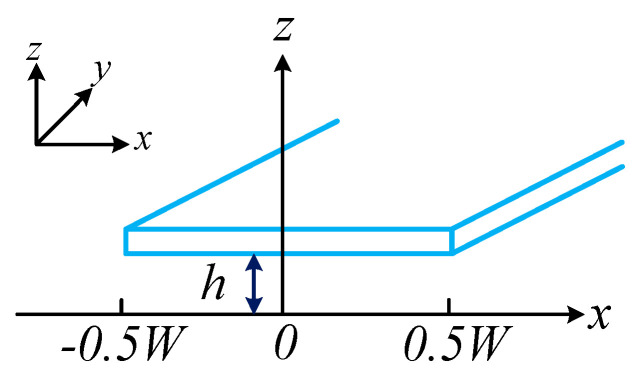
Schematic diagram of the squeeze film damping effect on a long rectangular plate.

In the context of CDRG, the air damping behavior of the drive mode is similar to that of the sense mode. Due to the gyroscope’s complex structure, performing finite element simulations of the squeeze film damping requires significant computational resources. To simplify the analysis, the structural model is approximated. The squeeze film movement between each ring and the fixed electrode is modeled as the squeeze film movement between a long rectangular plate and a fixed electrode. In this simplified model, the rectangular plate is assumed to have a specific length and width, as illustrated in Figure 3.

Since the squeeze film damping in the *Y* direction does not change, the Reynolds equation can be simplified to

(7) $$\frac{d^{2}P}{dx^{2}}=\frac{12\mu }{h^{3}}\frac{dh}{dt}$$

The boundary conditions are

(8) $$P\left(\pm \frac{1}{2}W\right)=0$$

To carry out the second integral, we obtain

(9) $$P(x,t)=\frac{6\mu }{h^{3}}\frac{dh}{dt}x^{2}+C_{1}x+C_{2}$$

The pressure distribution of the long rectangular plate can be obtained by using boundary conditions.

(10) $$P(x,t)=-\frac{6\mu }{h^{3}}\left(\frac{W^{4}}{4}-x^{2}\right)\frac{dh}{dt}$$

**Figure 4:**
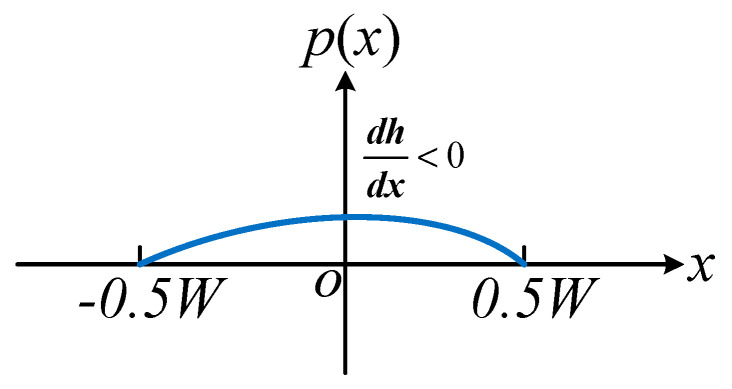
Pressure distribution diagram of squeeze film damping between long rectangular plates.

When the rectangular plate moves downward and the film is compressed (*dh*/*dt* < 0), the pressure becomes positive. At this moment, the pressure at x = 0 is the largest, 3 μW^4^/2 h^3^ (where *dh/dt* < 0); while the pressure at *x* = ±*W*/2 is 0. The pressure distribution is illustrated in Figure 4.

Damping force of a long rectangular plate is

(11) $$F_{lr}=\int_{-W/2}^{W/2}P(x)Ldx=-\frac{\mu W^{3}L}{h^{3}}\frac{dh}{dt}=-\frac{\mu W^{3}L}{h^{3}}\dot{h}$$

According to the definition $F=-c\dot{x}$, the damping coefficient of the long rectangular plate can be determined.

(12) $$c_{lr}=\frac{\mu W^{3}L}{h^{3}}$$

By substituting the relevant parameters into Equation (12), the squeeze film damping coefficient of CDRG can be derived as follows:

(13) $$C_{sq,C}=\frac{\mu H^{3}\sum_{i=1}^{n}A_{i}}{d_{0}^{3}}$$

where *H* is the thickness of the resonator, *n* is the number of rings, *A_i_* is the circumference of the vibration part corresponding to the *i*-th ring, and *d*_0_ is the gap between the resonator and the electrode. Therefore, the squeeze film damping quality factor *Q_sq,C_* is

(14) $$Q_{sq,C}=\frac{m_{eff}\omega_{n0}d_{0}^{3}}{\mu H^{3}\sum_{i=1}^{n}A_{i}}$$

where *m_eff_* is effective mass of the resonator, and *ω_n_*_0_ is angular frequency of the intrinsic mode.

In the discontinuous flow region, the viscosity coefficient *μ* can be corrected by the effective viscosity coefficient *μ_eff_*. For MEMS structures, Equation (5) can be applied in a correction to obtain more accurate data. By substituting the Formulas (3)–(5) into the Formula (14), we derive the final expression for *Q_sq,C_* as follows:

(15) $$Q_{sq,C}=\frac{m_{eff}\omega_{n0}d_{0}^{3}}{H^{3}\sum_{i=1}^{n}A_{i}}\frac{1+9.638{\left(\frac{BT}{pd_{0}}\right)}^{1.159}}{\mu_{0}}\frac{1+T_{s}/T}{1+T_{s}/T_{0}}\sqrt{\frac{T_{0}}{T}}$$

Given the parameter *T* = 298.15 K (in order to ensure the consistency with the experimental temperature, the following are set at room temperature of 298.15 K), *m_eff_* = 0.268 mg, $\sum_{i=1}^{n}A_{i}=0.0{31\;\mathrm{mm}}^{2}$, and *p* = 0.07 Pa, and according to Equation (15), the quality factor of the squeeze film damping in the modified continuous fluid model is 2.075 × 10^7^.

#### 3.1.2. Energy Transfer Model

**Figure 5:**
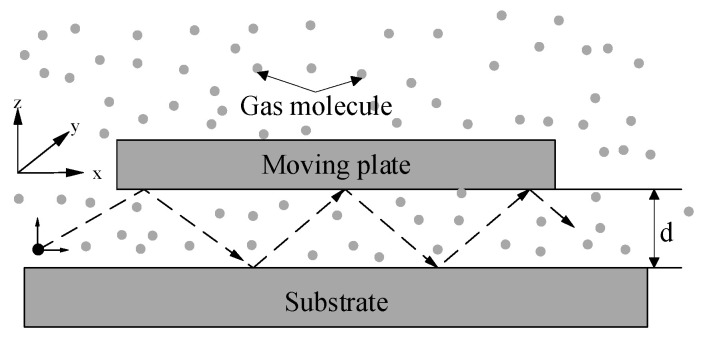
Gas particles between a substrate and a moving plate for the energy transfer model.

In extremely low-pressure environments (<1 Pa), collisions between gas molecules become infrequent, making it challenging to treat the gas as a viscous fluid. In such cases, the concept of effective viscosity may no longer apply. An energy transfer model, which describes the interactions between gas molecules and the vibrating plate during collisions, could provide a more accurate representation. To simplify the analysis of the MEMS cobweb-like disk resonant gyroscope, its structure is approximated as a rectangular plate, as illustrated in Figure 5.

To determine the analytical solution, it is essential to first consider the change in the velocity of gas molecules after colliding with the resonator [14]. It is assumed that the gas molecules collide with the resonator at a velocity *v*, and the velocity of the collision position on the resonator is $\dot{z}$. Since the mass of the gas molecular *M_m_* is much smaller than the resonator mass *M*, we can apply the principle of momentum conservation. Therefore, the gas molecular velocity after the collision is given by

(16) $$v_{+}=v+2\dot{z}$$

Similarly, if the resonator moves in the same direction as the gas molecules, the velocity of the gas molecules will be

(17) $$v_{+}=v-2\dot{z}$$

Next, we need to calculate the energy dissipated by the collision of gas molecules in a unit period. The gap between the resonator and the fixed electrode is denoted as *d*_0_. Assuming the vibration displacement of the resonator *z* = *z*_0_sin*ωt*, the gap becomes *d* = *d*_0_ − *z*_0_sin*ωt* during the vibration process. Considering the perimeter of the gas film boundary is *L*, the boundary area is

(18) $$S=L\left(d_{0}-z_{0}\cos\omega t\right)$$

The number of gas molecules passing through the boundary per unit time is

(19) $$N_{0}=\frac{1}{4}n_{c}\bar{v}S=\frac{1}{4}n_{c}\bar{v}L\left(d_{0}-z_{0}\cos\omega t\right)$$

where *n_c_* and $\bar{v}$ are the concentration and mean velocity of the gas molecules, respectively, and $\bar{v}=\sqrt{8kT/\pi M_{m}}$.

When the gas molecules enter the gap, the velocity component in the z direction is *v_z_*_0_, the velocity component in the XY plane is *v_xy_*_0_, and the transverse displacement is *l*, so the residence time in the gap is Δ*t* = *l*/*v_xy_*_0_. Since this is much less than the vibration period of the resonator [15], the collision time of the gas molecules in each vibration period is

(20) $$\Delta N=\frac{v_{z0}\Delta t}{2\left(d_{0}-z_{0}\sin\omega t\right)}=\frac{v_{z0}l}{2\left(d_{0}-z_{0}\sin\omega t\right)v_{xy0}}$$

When the gas molecules collide with the plate and the velocity increment is $2\dot{z}$, the *z*-direction velocity at the end of the movement in the gap is

(21) $$v_{z}=v_{z0}+\Delta N\cdot 2\dot{z}=v_{z0}+\frac{v_{z0}lz_{0}\omega \cos\omega t}{\left(d_{0}-z_{0}\cos\omega t\right)v_{xy0}}$$

The molecular potential energy of the gas inside the entry gap region is

(22) $$e_{k,in}=\frac{1}{2}M_{m}\left(v_{z0}^{2}+v_{xy0}^{2}\right)$$

The molecular potential energy of the gas in the escape gap region is

(23) $$e_{k,\mathrm{out}}=\frac{1}{2}M_{m}\left(v_{z}^{2}+v_{xy0}^{2}\right)$$

Therefore, the additional energy obtained by the gas molecules through the collision with the resonator is

(24) $$\Delta e_{k}=e_{k,\mathrm{out}}-e_{k,in}=\frac{1}{2}M_{m}\left[\frac{2v_{z0}^{2}lz_{0}\omega \cos\omega t}{\left(d_{0}-z_{0}\cos\omega t\right)v_{xy0}}+\frac{v_{z0}^{2}l^{2}z_{0}^{2}\omega^{2}\cos^{2}\omega t}{{\left(d_{0}-z_{0}\cos\omega t\right)}^{2}v_{xy0}^{2}}\right]$$

It is evident that the additional energy acquired by the gas molecules originates from the resonator. Since the average value of the first term on the right side of the equation is zero over one vibration period of the resonator, only the second term contributes to the energy dissipation of the resonator. By combining Equations (19) and (24), the average energy dissipation of the resonator during one vibration period can be expressed as

(25) $$\Delta E_{cycle}=\int_{0}^{2\pi }\frac{n_{c}\bar{v}LM_{m}v_{z0}^{2}l^{2}z_{0}^{2}\omega \cos^{2}\omega t}{8\left(d_{0}-z_{0}\cos\omega t\right)v_{xy0}^{2}}d\omega t$$

To predict the calculation, *l*^2^, $v_{z0}^{2}$ and *z*_0_ are approximated by $\bar{l^{2}}$, $\bar{v_{z0}^{2}}$ and $\bar{z_{0}}$, respectively. Since ${\bar{v}}_{xy0}^{2}=2{\bar{v}}_{z0}^{2}$ [16], the Formula (25) can be converted into

(26) $$\Delta E_{cycle}=\int_{0}^{2\pi }\frac{\rho_{o}\bar{v}L\bar{l^{2}}{\bar{z_{0}}}^{2}\omega \cos^{2}\omega t}{16\left(d_{0}-\bar{z_{0}}\cos\omega t\right)}d\omega t$$

where *ρ_0_* = *n_c_M_m_* is the gas density. Assuming that the vibration shift *z* of the resonator is much less than the gap *d*_0_, there is

(27) $$\Delta E_{cycle}\approx \frac{\pi \rho_{o}\bar{v}L\bar{l^{2}}{\bar{z_{0}}}^{2}\omega }{16d_{0}}$$

Hence, the quality factor *Q_sq,e_* for squeeze film damping, based on the energy transfer model at low pressure, is expressed as

(28) $$Q_{sq,E}=\frac{2\pi E_{v}}{\Delta E_{cycle}}=\frac{16m\omega d_{0}}{\rho_{o}\bar{v}L\bar{l^{2}}}$$

where *E_v_* and m are the vibration energy and mass of the resonators, respectively. According to the energy transfer theory, $\bar{l^{2}}=2A/\pi$, A is the overlapping area of the resonator ‘s vibration part and the fixed electrode, *ρ*_0_ = *M_m_p*/*RT*, $\bar{v}=\sqrt{8RT/\pi M_{m}}$; then, *Q_sq,e_* can be expressed as

(29) $$Q_{sq,E}={\left(2\pi \right)}^{\frac{3}{2}}\frac{m\omega d_{0}}{LA}\sqrt{\frac{RT}{M_{m}}}\frac{1}{p}$$

By substituting the structural parameters of the CDRG, the final expression for *Q_sq,e_* can be obtained as follows:

(30) $$Q_{sq,E}={\left(2\pi \right)}^{\frac{3}{2}}\frac{m\omega d_{0}}{2\sum_{i=1}^{n}\left(A_{i}+H\right)\sum_{i=1}^{n}A_{i}H}\sqrt{\frac{RT}{M_{m}}}\frac{1}{p}$$

**Figure 6:**
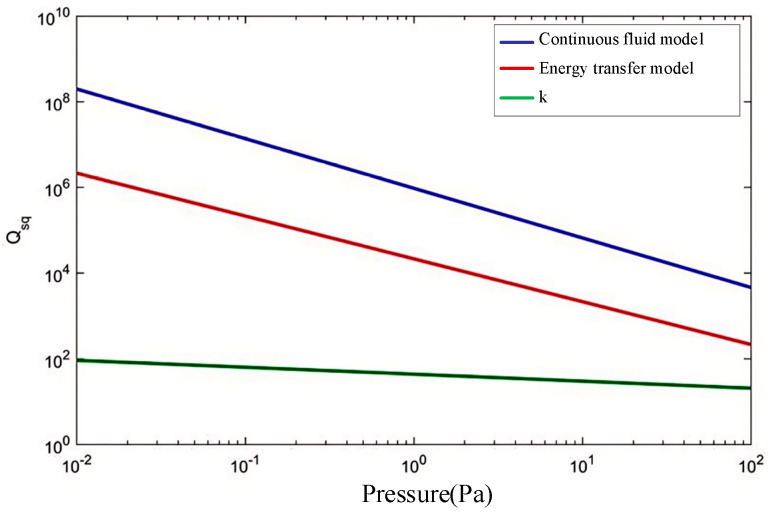
The relationship between the damping of the two models and the pressure, and *k* is the error coefficient.

Based on the provided parameters, the calculations from the energy transfer model are presented in Table 2. A comparison of the quality factor curves for the two models with respect to pressure is illustrated in Figure 6. It is evident that as the pressure decreases, the error increases for both models. This may be attributed to the presence of air molecules in high-vacuum conditions, where they can no longer be considered a continuous fluid. Consequently, the energy transfer model may provide a more accurate representation, which will be confirmed through experiments discussed in the following sections.

### 3.2. Thermoelastic Damping

#### 3.2.1. The Thermoelastic Damping Loss Mechanism

When a MEMS device experiences vibration, material strain results in irreversible energy dissipation, known as thermoelastic damping (TED). For MEMS devices operating in ultra-high-vacuum conditions, TED is the dominant mechanism limiting their quality factor [17]. The thermomechanical coupling process responsible for TED is described as follows.

Thermal expansion, a well-established phenomenon, occurs when temperature variations induce dimensional changes in solids. This interaction between mechanical and thermal fields is bidirectional. Specifically, a solid under tensile stress will experience temperature changes: compressive strain typically causes a temperature increase, while tensile strain results in a temperature decrease, particularly in materials like silicon.

Two mechanisms explain the thermal dissipation process. One explanation posits that the strain gradient in a bending beam induces a corresponding temperature gradient. As heat flow mitigates this temperature gradient, thermoelastic dissipation occurs, irreversibly converting vibrational energy into heat. Alternatively, mechanical energy can generate a temperature gradient, resulting in energy exchange between the system and entropy. Heat flow naturally attempts to balance the temperature gradient, leading to irreversible energy dissipation in the form of increased entropy.

While TED cannot be entirely eliminated in any resonator, it can be minimized through structural optimization and material selection. Based on the coupling between the strain field and the temperature field, TED models can be classified into two categories. The first category considers unidirectional coupling, where the strain field influences the temperature field, but not vice versa; a typical example is the Zener analytical model. The second category involves full coupling, where both strain and temperature fields influence each other, as exemplified by the COMSOL 6.2 simulation model. The first model is simpler and computationally efficient, while the second model, although more complex, offers greater accuracy.

**Figure 7:**
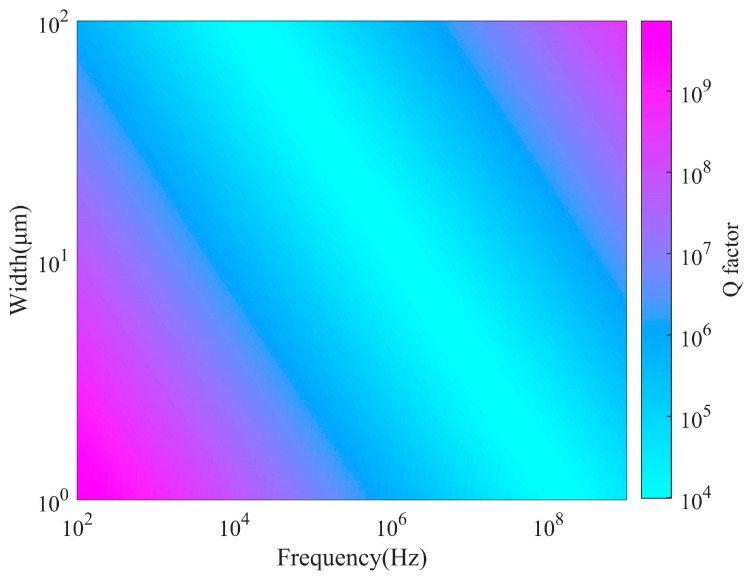
The relationship between *Q_TED_*, ring width, and vibration frequency.

Subsequently, estimations will be performed using both models. The methods of mode superposition and separation of variables will be employed, along with the introduction of triangular series to satisfy the boundary conditions, leading to the final expression [18]:

(31) $$Q_{TED,Zener}=\frac{1}{\Delta_{E}}\frac{1+{\left(\omega_{0}\tau \right)}^{2}}{\omega_{0}\tau }=\frac{\rho C_{\rho }}{E\alpha^{2}T_{0}}\frac{1+{\left(\omega_{0}\tau \right)}^{2}}{\omega_{0}\tau }$$

where *ω*_0_ is the natural frequency and *τ* = *W*^2^*ρ*C_p_/kπ^2^ is the thermal relaxation time. Obviously, the quality factor *Q_TED_* is partially dependent on the material properties, which may also vary with ambient temperature *T*_0_. Additionally, the thermal relaxation time *τ* is related to the heat transfer rate across the entire cross-section of the resonant beam and depends on the beam width *W* (which defines the length of the heat flow path). Furthermore, the natural frequency *ω*_0_ is influenced by both the size of the beam and its material characteristics. The function $\left(1+\omega_{0}^{2}\tau^{2}\right)/\omega_{0}\tau$ describing Debye behavior reaches a minimum at the frequency *ω*_0_ = *ω_min_* = 1/*τ*. Therefore, the *Q_TED_* of the resonator changes significantly depending on the proximity of the natural frequency *ω*_0_ to *ω_min_*. When *ω*_0_ = *ω_min_*, *Q_TED_* takes the minimum value, corresponding to the maximum thermoelastic damping; when *ω*_0_ < *ω_min_*, the heat transfer time constant is shorter than the vibration period, making the system effectively isothermal. Conversely, when *ω*_0_ > *ω_min_*, the heat transfer time constant exceeds the vibration period, rendering the system adiabatic. Table 3 lists the material properties of (111) monocrystalline silicon, while Figure 7 illustrates the relationship between *Q_TED_*, the ring width (beam width) and frequency. The color bar in the figure represents the magnitude of the *Q* value. For a MEMS gyroscope with a ring width of 13 μm, the analytical solution for *Q_TED_* using the Zener model is 2.452 × 10^5^ at room temperature (*T* = 298.15 K).

#### 3.2.2. COMSOL Finite Element Model

The COMSOL simulation model considers the anisotropic thermoelastic coupling in solid structures, where the interaction between stress and temperature fields exhibits a full coupling effect. The 3D elastic solid motion equation and the isotropic thermoelastic solid constitutive equation are combined [19,20], and the simplified tensor method is applied to obtain the displacement linear equation as follows:

(32) $$\rho \frac{\partial^{2}u}{\partial t^{2}}=\mu \left(\frac{\partial^{2}u}{\partial x^{2}}+\frac{\partial^{2}u}{\partial y^{2}}+\frac{\partial^{2}u}{\partial z^{2}}\right)+\left(\lambda +\mu \right)\left(\frac{\partial^{2}u}{\partial x^{2}}+\frac{\partial^{2}v}{\partial x\partial y}+\frac{\partial^{2}w}{\partial x\partial z}\right)-\gamma \frac{\partial T}{\partial x}$$

(33) $$\rho \frac{\partial^{2}v}{\partial t^{2}}=\mu \left(\frac{\partial^{2}v}{\partial x^{2}}+\frac{\partial^{2}v}{\partial y^{2}}+\frac{\partial^{2}v}{\partial z^{2}}\right)+\left(\lambda +\mu \right)\left(\frac{\partial^{2}u}{\partial y\partial x}+\frac{\partial^{2}v}{\partial y^{2}}+\frac{\partial^{2}w}{\partial y\partial z}\right)-\gamma \frac{\partial T}{\partial y}$$

(34) $$\rho \frac{\partial^{2}w}{\partial t^{2}}=\mu \left(\frac{\partial^{2}w}{\partial x^{2}}+\frac{\partial^{2}w}{\partial y^{2}}+\frac{\partial^{2}w}{\partial z^{2}}\right)+\left(\lambda +\mu \right)\left(\frac{\partial^{2}u}{\partial z\partial x}+\frac{\partial^{2}v}{\partial z\partial y}+\frac{\partial^{2}w}{\partial z^{2}}\right)-\gamma \frac{\partial T}{\partial z}$$

where *u*, *v*, and *w* are the displacement in *x*, *y*, and *z*, respectively, and the thermoelastic coefficient γ = *α*(3*λ* + 2*μ*), *λ* = *E*/(1 + *v*) (1 − 2*v*) and *μ* = *E*/2(1 + *v*) is the Ramer coefficient (Lamé parameters). Through Fourier’s law of entropy and the constitutive matrix equation, the thermal coupled linear equation of the temperature field is obtained as [21] follows:

(35) $$\kappa \nabla^{2}T=\rho C_{p}\frac{dT}{dt}+T_{0}\gamma \left(\frac{\partial \dot{u}}{\partial x}+\frac{\partial \dot{v}}{\partial y}+\frac{\partial \dot{w}}{\partial z}\right)$$

Formulas (32) to (35) above form a set of 3D thermoelastic coupled linear equations, where the coupling between structural stress and temperature fields is governed by the thermal expansion coefficient α. Due to their linear nature, these equations can be solved for various geometries using the finite element method (FEM). COMSOL finite element simulation software, equipped with built-in thermoelastic and solid mechanics physics modules, facilitates the coupling of heat transfer processes between stress and temperature fields, making it suitable for simulation calculations. In this model, thermal insulation boundary conditions are applied to all external surfaces.

**Figure 8:**
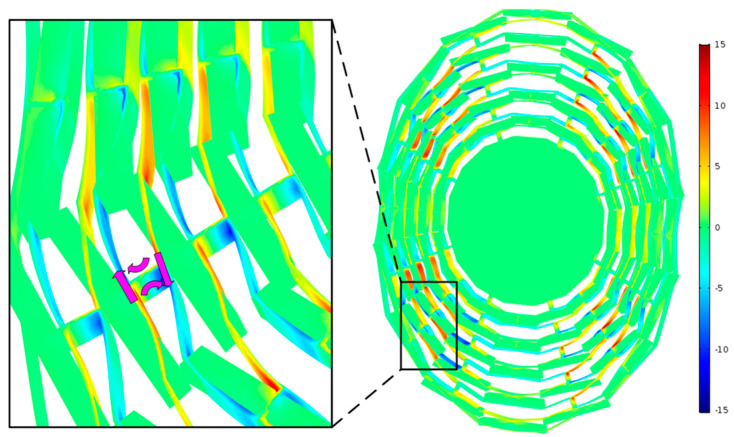
The temperature gradient distribution in the working mode of CDRG, with the purple arrows indicating the heat flow path.

The thermoelastic damping of the MEMS cobweb-like disk resonator gyroscope was simulated using COMSOL 6.2, with the temperature gradient distribution of the operational mode shown in Figure 8. Areas with higher temperatures (in the compressive stress zones) are depicted in red, while those with lower temperatures (in the tensile stress zones) are shown in blue. The purple arrows illustrate the direction of heat flow.

From a macroscopic viewpoint, the mass block suspended within the structural frame does not substantially contribute to heat dissipation, resulting in the overall structure remaining near thermal equilibrium, with only slight temperature deviations. However, a localized magnified view reveals that the temperature gradient is concentrated in areas with significant deformation, such as the rings and spokes, where temperature deviations are most pronounced.

The final quality factor value obtained from the COMSOL simulation at room temperature (*T* = 298.15 K) is 1.733 × 10^5^, as shown in Table 4. Compared to the analytical solution derived from the Zener model, this result displays a numerical difference of 41.5%, indicating a notable discrepancy. This variation arises due to the complex geometry of the structure, which deviates from a simple beam configuration, resulting in considerable coupling between the stress and temperature fields. Consequently, the Zener model alone may not provide precise results, making the FEM approach essential for achieving higher accuracy.

### 3.3. Anchor Loss

In MEMS structures, all resonant elements are fixed to a substrate, with the connection point between the resonant structure and the substrate known as the anchor. The energy dissipation mechanism, where part of the vibrational energy radiates as an elastic wave from the anchor and dissipates into the substrate, is referred to as anchor damping loss [22]. This loss is influenced by multiple factors, including the structural form, vibrational state, and the size and position of the support structure.

For simple resonant beams with well-defined vibration characteristics, anchor damping can be calculated using analytical models [23]. However, for complex axisymmetric structures like the cobweb-like disk resonant gyroscope, establishing an analytical model for anchor damping is highly challenging. Therefore, COMSOL finite element analysis (FEA) is employed for simulation.

A primary difficulty in simulating anchor damping arises from the nature of the substrate, which behaves as a semi-infinite medium. Elastic waves propagating into the substrate continue indefinitely without reflecting off sidewalls, complicating the modeling process. To address this, the substrate is represented as a finite domain surrounded by artificial boundaries that mimic the behavior of an infinite substrate.

One key characteristic of an infinite substrate is that waves entering it do not reflect back from the boundary. To approximate this behavior in a finite domain, non-reflective boundaries are often applied; however, this method is effective only when the incident wave is perfectly perpendicular to the boundary. To overcome this limitation, a more effective solution is to implement a Perfectly Matched Layer (PML), which is a specialized dielectric layer that matches the wave impedance of the adjacent medium. The PML allows incident waves to pass through the boundary without reflection and attenuates the waves, effectively meeting the requirements for accurate modeling.

In this simulation, a PML is added to the outer boundary of the model’s substrate to prevent elastic waves from reflecting back and distorting the results. The PML enhances wave damping by converting the wave solution into a complex coordinate system within the PML region, resulting in an exponential decay of the wave amplitude. If the PML region is sufficiently large, the exponential decay ensures that the wave diminishes to negligible levels. Even if some attenuated waves reflect from the PML boundary, their energy upon returning to the resonator is minimal, making their impact negligible.

To calculate the *Q* factor for anchor damping, the PML order and scaling factor were set to 1. Depending on the resonator design and boundary conditions, Cartesian, cylindrical, or spherical PMLs can be utilized to absorb stress waves. Since spherical PMLs are particularly effective at absorbing radial waves, they were selected for this simulation.

In this study, we assume that the gyro resonators, anchors, substrate, and Perfectly Matched Layers (PMLs) are constructed from (111) monocrystalline silicon, with the material properties detailed in Table 3. The quality of the grid cells and the dimensions of the PMLs are critical factors when calculating the quality factor (*Q* values) using PMLs. Poor grid quality can lead to inadequate convergence of iterative solvers and pathological behaviors in the solution process. Generally, increasing the number of grid cells enhances numerical accuracy but also raises solution time and memory requirements.

**Figure 9:**
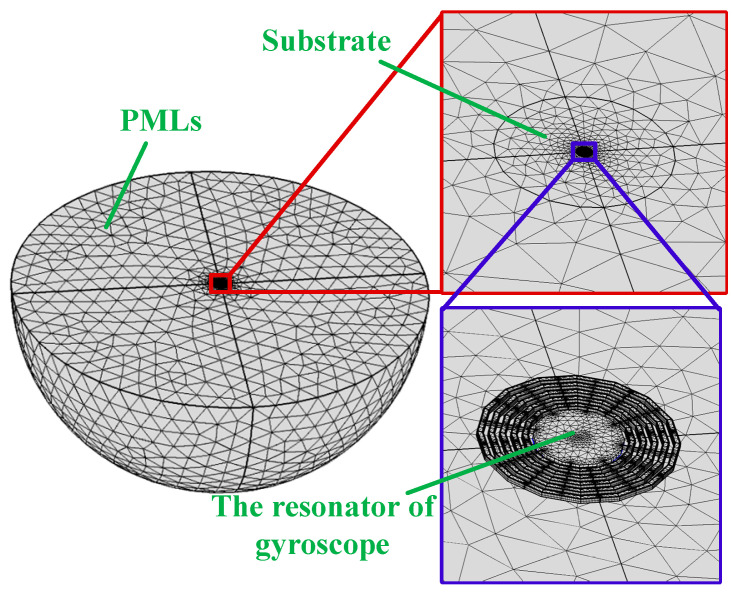
The grid dissection results of PMLs for a CDRG.

Given practical constraints, we prioritize grid accuracy for the gyroscope resonator structure. The meshing process begins with a swept approach for the resonator surface, followed by the creation of a free tetrahedral mesh for both the substrate and the PML. The results of the final grid dissection are illustrated in Figure 9.

To ensure accurate simulation results, the PML radius was set to match the mechanical wavelength during the analysis. For longitudinal waves propagating through the solid material, the mechanical wavelength is defined by the following expression [24]:

(36) $$\lambda =\frac{1}{f}\sqrt{\frac{E\left(1-\upsilon \right)}{\rho \left(1+\upsilon \right)(1-2\upsilon )}}$$

where *f* is the resonator’s vibration frequency. The MEMS cobweb-like disk axisymmetric gyroscope resonance frequency design value is 18,188 Hz, as shown in Table 3, and it is calculated that *λ* = 4.72 × 10^5^ μm. This value is used to define the edge length of the Perfectly Matched Layer (PML). The quality factor *Q_anchor_* of the anchor damping can be expressed as follows [25]:

(37) Qanchor=Reω2Imω

where *Re*(*ω*) and *Im*(*ω*) represent the real and imaginary parts of the gyroscopic resonance angular frequency, respectively. Using this equation, the COMSOL finite element method numerically solves the intrinsic frequency problem via the characteristic frequency method within the solid mechanics module. The calculated quality factor *Q_anchor_* for anchor damping in the axisymmetric gyroscope of the CDRG is found to be 1.921 × 10^8^. This value is significantly higher than the quality factors associated with air damping and thermoelastic damping, indicating that anchor damping is largely negligible in the overall energy dissipation of the system.

### 3.4. Other Dissipation Mechanisms

#### 3.4.1. Surface Loss

Due to the MEMS fabrication process, the dimensions of the resonator decrease, resulting in an increased surface-to-volume ratio that amplifies the surface effects [26]. The resonator surface may contain impurities, lattice defects, adsorbates, and other imperfections, leading to surface stress and energy dissipation. Several researchers have analyzed this phenomenon of surface loss by exploring specific physical mechanisms and developing a range of models to explain the surface effects. Their studies indicate that surface loss is influenced by material properties as well as the presence of metals on the resonator surface.

For instance, Seoanez et al. established a “unified model” for surface loss mechanisms, which considers both the second-order energy system of the surface and the total resonator energy [27]. Additionally, Esashi and Yasumura et al. investigated the *Q* value of ultrathin MEMS resonant beams, demonstrating that the *Q* value is dependent on both the film thickness and the thickness of the surface oxide layer formed during thermal processing. In both cases, the *Q* value fluctuates significantly during the high-to-normal-to-high temperature cycle [28]. When the vibrational displacement is much smaller than the structural size, surface loss can be modeled using the complex variable form of Young’s modulus [29], that is, *E_C_* = *E* + *iE_d_*, where *E* is the conventional Young’s modulus and *E_d_* is Young’s modulus responsible for energy dissipation. The total energy of the resonant beam during vibration can be expressed as

(38) $$W=\frac{1}{6}bhE\int \varepsilon_{\max}^{2}\left(x\right)dx$$

Among these parameters, *b* is the beam width, h is the beam thickness, and *ε_max_* is the maximum surface strain experienced during vibration. If the thickness of the surface layer is denoted as *δ*, the surface energy dissipation per unit vibration period can be expressed as [30]

(39) $$\Delta W=2\pi \delta E_{d}\left(b+\frac{h}{3}\right)\int \varepsilon_{\max}^{2}\left(x\right)dx$$

Thus, the *Q* value of the surface loss is

(40) $$Q=\frac{bh}{3b+h}\frac{E}{2E_{d}\delta }$$

As illustrated by Equation (40), a decrease in structural thickness, along with increases in both the surface layer thickness and Young’s modulus, leads to a reduction in the *Q* value associated with surface loss. Consequently, for structures characterized by a high surface-to-volume ratio, such as thin micro-hemispherical geometries, surface loss frequently emerges as a significant contributor to energy dissipation. However, in the case of the CDRG under investigation, the thickness-to-diameter ratio is relatively large. This results in a surface loss quality factor exceeding 1 × 10^10^, rendering surface loss effects negligible for this particular structure.

#### 3.4.2. Electronic Damping

When the conductive surface exhibits finite resistivity, the movement of charges encounters resistance, leading to energy dissipation known as electronic damping. In the MEMS gyroscope structure, gyro resonance is excited by applying an AC voltage to the excitation electrode. The output signal from the detection capacitance is then amplified using a basic amplification circuit. Importantly, the voltage bias necessary for generating the readout signal also applies a force to the gyro resonator, resulting in electronic damping losses. Unlike the energy dissipation mechanisms discussed previously, electronic damping is independent of the gyro structure’s design and fabrication, instead being linked to the electronic components utilized in the experimental setup.

**Figure 10:**
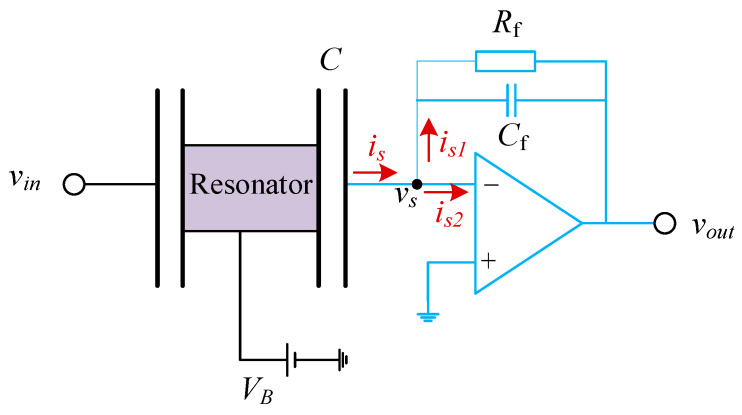
A simplified schematic diagram of the circuit model for the experimental setup of the CDRG.

For the CDRG, a charge amplifier serves as the pre-detection circuit module, as illustrated in Figure 10. The blue curve in the figure is the charge amplifier module, *v_in_* is the excitation AC voltage, *V_B_* is the offset DC voltage, *C* is the gyro detection capacitor, vs. is the gyro output voltage signal, and *R_f_* and *C_f_* are the feedback resistance and capacitance of the charge amplifier, respectively. According to Figure 10, the resonant vibration of the gyroscope can be described by the control equation of a second-order mechanical system, with the electrostatic force generated by two electrodes, as illustrated below [31].

(41) $$m_{eff}\ddot{x}+c\dot{x}+kx=\frac{{\left(V_{B}-v_{in}\right)}^{2}}{2}\frac{dC}{dx}+\frac{{\left(V_{B}-v_{s}\right)}^{2}}{2}\frac{dC}{dx}$$

where *c* and *k* are the damping and stiffness coefficients of the gyroscope, respectively.

To determine the output voltage vs. of the gyroscope, the current *i_s_* is calculated first. In an ideal operational amplifier, the gain is considered infinite, and the node voltage equals the input voltage. However, in practice, the amplifier gain is finite. By applying Kirchhoff’s current law and Ohm’s law, the following relationships can be established:

(42) $$i_{s}=i_{s1}+i_{s2}$$

(43) $$i_{s}=\left(V_{B}-v_{s}\right)\frac{dC}{dt}$$

(44) $$i_{s1}=\frac{v_{s}+Gv_{s}}{Z_{f}}$$

(45) $$i_{s2}=\frac{v_{s}}{Z_{in}}$$

where *Z_in_* is the input impedance of the amplifier, *G* is its amplification gain, *Z_f_* = *R_f_*_/_(1 + *sR_f_C_f_*) is the feedback impedance, and s is the complex frequency. Equations (46)~(50) are combined and expanded using Taylor series, as follows:

(46) $$m\ddot{x}+c\dot{x}+kx=\frac{{\left(V_{B}-v_{in}\right)}^{2}}{2}\frac{dC}{dx}+\frac{V_{B}^{2}}{2}\frac{dC}{dx}-Z_{s}V_{B}^{2}{\left(\frac{dC}{dx}\right)}^{2}\dot{x}+\frac{Z_{s}^{2}V_{B}^{2}}{2}{\left(\frac{dC}{dx}\right)}^{3}{\dot{x}}^{2}$$

where

(47) $$Z_{s}=\frac{1}{\frac{1}{Z_{in}}+\frac{\left(G-1\right)\left(1+sR_{f}C_{f}\right)}{R_{f}}}$$

The third term on the right side of Equation (51) represents the damping effect due to the experimental electronics, as it is proportional to the velocity. Utilizing the relationship between damping and *Q* value, the electronic damping quality factor *Q_elec_* can be derived as follows:

(48) $$Q_{elec}=\frac{2\pi m_{eff}f}{V_{B}^{2}Z_{s}{\left(\frac{dC}{dx}\right)}^{2}}$$

Equation (48) indicates that *Q_elec_* increases as the equivalent output impedance of gyroscope *Z_s_* and the bias DC voltage *V_B_* decrease. The *Q_elec_* for the study object has been calculated using the parameters listed in Table 5. Based on these parameters, the calculated *Q_elec_* is 3.08 × 10^10^. Clearly, the contribution of electronic damping is negligible compared to that of the other damping mechanisms.

#### 3.4.3. Akhiezer Damping

The energy dissipation caused by interactions between elastic waves and thermal phonons within a material is categorized as an intrinsic damping loss mechanism. This mechanism can be divided into two components: spatial phonon transport and local phonon scattering. Irreversible spatial phonon transport, resulting from time-varying strain gradients, corresponds to the previously examined thermoelastic damping loss mechanism. In contrast, local phonon scattering, first identified by Akhiezer in 1939, is known as Akhiezer damping. For high-frequency bulk mode resonators, Akhiezer damping is often the primary factor limiting the quality factor, whereas in low-frequency resonators, it is typically negligible compared to thermoelastic damping.

Woodruff, applying the linear Boltzmann transport equation and the Debye model, derived a simplified expression for isotropic Akhiezer damping [32], which considered only classical properties. Subsequently, Iyer et al. explored the anisotropic energy storage and dissipation due to non-harmonic phonon–phonon interactions in cubic semiconductors and dielectric crystals, deriving an expression for the Akhiezer damping quality factor [33]. The following equation represents the quality factor for Akhiezer damping [34].

(49) $$f\cdot Q_{Akhiezer}=\frac{3\rho c^{2}c_{D}^{2}}{2\pi \gamma_{eff}^{2}\kappa T}$$

where *c* is the acoustic wave velocity, *c_D_* is the Debye velocity, and *γ_eff_* is the effective Gruneisen parameter. The Grüneisen parameter, which depends on the mode pattern and material anisotropy, is typically treated as a free parameter for fitting. For silicon materials, the order of *f* · *Q_Akhiezer_* is near 1 × 10^13^. Using this relationship, the *Q_Akhiezer_* of the CDRG is estimated to be approximately 1 × 10^9^.

In conclusion, the quality factors for surface damping, electronic damping, and Akhiezer damping are all on the order of 1 × 10^8^, making them negligible compared to the air damping and thermoelastic damping mechanisms. Table 6 presents the theoretical results and contribution rates for the total quality factor and each damping mechanism. Notably, the thermoelastic damping quality factor is derived solely from finite element analysis.

From Table 6, the results calculated using different air damping models show significant variation in the total quality factor and the contribution rates of the primary damping mechanisms. When the continuous fluid model is applied, the total quality factor is approximately 1.716 × 10^5^, with the thermoelastic damping mechanism being dominant, contributing 99.07%. This indicates that the overall quality factor is mainly influenced by thermoelastic damping. In contrast, using the energy transfer model yields a total quality factor of about 1.105 × 10^5^, where both thermoelastic and air damping mechanisms are significant, contributing 63.79% and 36.14%, respectively. Accurate experimental measurements are needed to determine which air damping model provides a more precise representation.

## 4. Verification and Discussion

A detailed theoretical analysis of the damping loss mechanism has been previously discussed. To validate this theoretical analysis, experimental testing of the fabricated prototype is necessary. The following section provides experimental verification of the energy dissipation mechanism model and the damping asymmetry error mechanism.

Two common methods exist for measuring the quality factor (*Q*): the frequency response method and the time decay method. For high-*Q* resonators, the −3 dB bandwidth is very narrow, making the measurement of *Q* values using the frequency response method increasingly difficult and prone to error. As a result, the time decay method is used for measuring the *Q* value of the resonator. Initially, the resonant frequency is determined using the sweep method, and the resonator is then excited at this frequency. After allowing the oscillation to reach a stable state, the excitation signal from the signal generator is turned off, and the decay oscillation data are collected using the NI data acquisition card. The Hilbert transform is then applied to extract the envelope of the signal, and the time constant *τ* and *Q* are derived by fitting an exponential decay model. The time required for the amplitude to decay to 1/*e* of its initial value is defined as the time constant *τ*, from which the quality factor *Q* can be calculated using the following formula:

(50) $$Q=\pi f_{0}\tau$$

where *f*_0_ is the resonant frequency.

**Figure 11:**
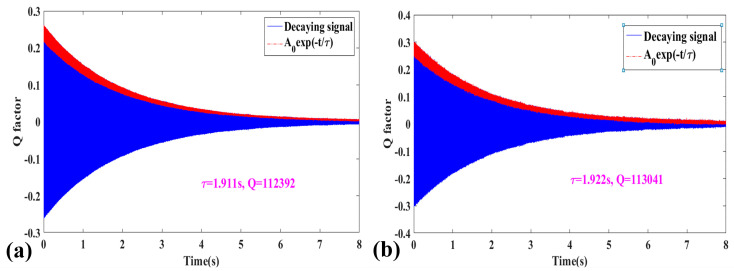
Quality factor test results of CDRG # 1 at room temperature (*T* = 298.15 K). (**a**) Drive mode; (**b**) sense mode.

The time constant *τ* and *Q* values of the drive mode for CDRG #1 are 1.911 s and 1.124 × 10^5^, respectively, while those for the sense mode are 1.922 s and 1.13 × 10^5^, as illustrated in Figure 11. In Figure 11, the blue segment represents the attenuation signal, and the red segment corresponds to the *A*_0_ exp(−*t*/*τ*) fitted curve. The *Q*-value test results for the remaining seven CDRG prototypes are summarized in Table 7. According to the test data, the quality factor of the CDRG prototypes ranges from 0.66× 10^5^ to 1.23 × 10^5^.

**Figure 12:**
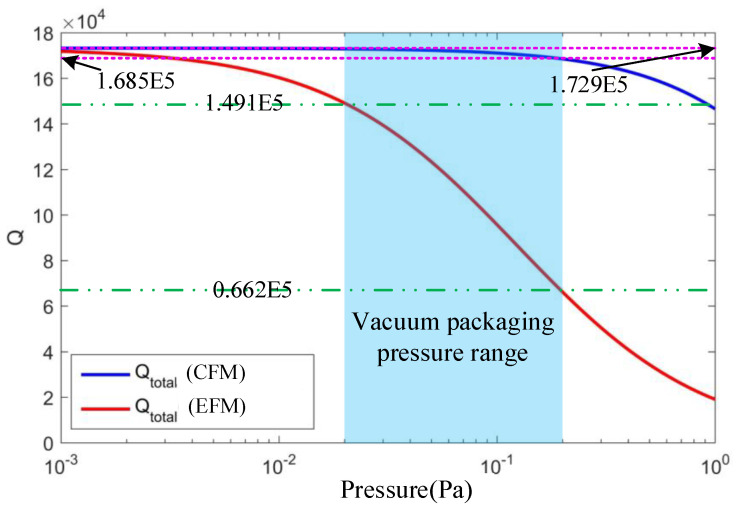
The relation between the total energy dissipation mechanism of the modified continuous fluid model (CFM) and the energy transfer model (EFM) at room temperature (*T* = 298.15 K).

In Figure 12, the theoretical analysis results comparing the relationship between the total quality factor based on the modified continuous fluid model (CFM) and that based on the energy transfer model (EFM) with pressure and at room temperature (*T* = 298.15 K) are presented. According to previous studies on the damping loss mechanisms of vibrating ring gyros, the pressure range for wafer-stage vacuum packaging is approximately 0.02 Pa to 0.2 Pa, influenced by the adsorption effect of the getter, which is the light blue area in Figure 12. In the corresponding pressure region, the total quality factor for the CFM ranges from 1.685 × 10^5^ to 1.729 × 10^5^ (see the purple dotted line in Figure 12), while the quality factor for the EFM spans from 0.662 × 10^5^ to 1.491 × 10^5^ (see the green dotted line in Figure 12).

Since the test results of the quality factor for the CDRG prototypes ranges from 0.66 × 10^5^ to 1.23 × 10^5^, it is obvious that these results are within the range of the theoretical analysis results for the total quality factor based on the EFM, while it is outside the range for the total quality factor based on the CFM, so the theoretical model of the total energy dissipation mechanism based on the EFM is more consistent with the experimental results.

Furthermore, the theoretical model of the total energy dissipation mechanism loss mechanism based on the energy transfer model can be further validated through a temperature characteristic test of the quality factor. The temperature characteristics of the total energy dissipation mechanism are primarily influenced by both the air damping loss mechanism and the thermoelastic damping loss mechanism. To describe the temperature dependence of the quality factor, the temperature coefficient (temperature coefficient of quality factor, TCQ) is introduced. Typically, the TCQ is expressed in an exponential form, such as *Q*∝T^−TCQ^.

**Figure 13:**
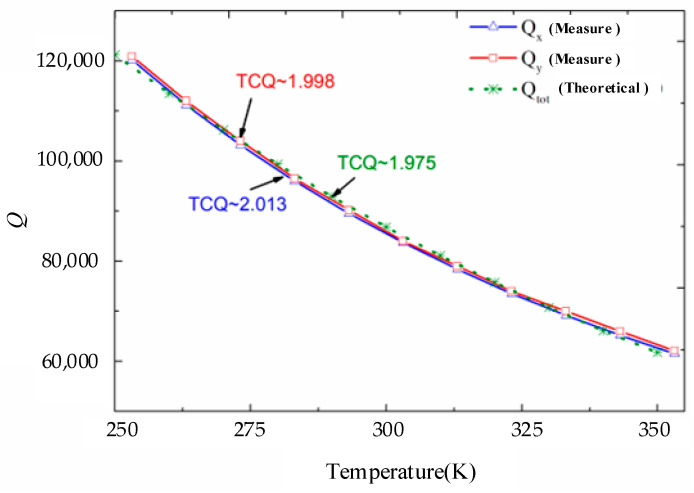
Comparison of measured results and theoretical models of device CDRG # 3.

Due to the damage sustained by device CDRG #1 after multiple uses, device CDRG #3 was employed to evaluate the quality factor and temperature characteristics. The time decay method was used for the measurement, and the results are presented in Figure 13. In Figure 13, the blue and red curves represent the drive mode quality factor and the sense mode quality factor of device CDRG #3, with TCQs of 2.013 and 1.998, respectively.

Based on the measured results for the quality factor of CDRG #3, the energy transfer model estimates that, at a packaging pressure of approximately 0.12 Pa, the damping quality factor is around 1.784 × 10^5^. In reference [35], it is noted that the quality factor for the air damping loss mechanism is expressed as *Q_air_*∝*T*^−0.5^, indicating that its temperature coefficient is the opposite. This discrepancy arises because the device is vacuum-packed, meaning the volume remains constant while the pressure varies as a function of the temperature. Thus, the combination of Equation (30) and the ideal gas state equation can be expressed as follows:

(51) $$Q_{sq,e}={\left(2\pi \right)}^{\frac{3}{2}}\frac{m\omega d_{0}}{2\sum_{i=1}^{n}\left(A_{i}+H\right)\sum_{i=1}^{n}A_{i}H}\sqrt{\frac{1}{M_{m}RT}}\frac{V}{n}$$

In Equation (51), *V* represents the gas volume, and *n* denotes the amount of substance in the wafer-level package. These values are fixed and independent of temperature for the packaging device, and the quality factor for the air damping is also given by *Q_air_*∝*T*^−0.5^. In addition, the thermoelastic damping is very sensitive to temperature. In the finite element model, (111) single-crystal silicon is utilized, and the TCQ for thermoelastic damping is approximately 3.5, consistent with findings reported in the literature [35].

Finally, using the energy transfer model and finite element analysis, we derived the theoretical temperature characteristic curve for the total energy dissipation mechanism, illustrated by the green dashed line in Figure 13. This curve indicates a TCQ of approximately 1.975. When compared to the experimental results, the temperature coefficient is closely aligned, with a maximum error not exceeding 2%. These findings demonstrate that the theoretical model is generally consistent with the experimental results and can effectively estimate the loss mechanisms of the gyro structure, providing significant guidance for its design and optimization.

## 5. Conclusions

In this paper, we systematically establish a theoretical model for the energy dissipation mechanisms of the MEMS cobweb-like disk resonator gyroscope, which includes air damping, thermoelastic damping, anchor loss, and other losses. Air damping in the free molecular flow regime is analyzed using both a continuous fluid model and an energy transfer model. The results obtained from the theoretical model using the energy transfer approach align well with the measured data, with the maximum error in the temperature coefficient being less than 2%. This discrepancy may arise from fabrication errors and measurement inaccuracies. The results indicate that thermoelastic damping and air damping are the primary energy dissipation mechanisms in the MEMS cobweb-like disk resonator gyroscope, accounting for 63.79% and 36.14%, respectively. In contrast, anchor loss accounts for about 5.8%, while other losses, including surface loss, electronic damping, and Akheizer damping, contribute only about 1.1%. This suggests that even with high-vacuum packaging, air damping continues to significantly impact the quality factor when the capacitor gap is small. Therefore, to enhance the quality factor of resonator structures with vacuum packaging, it is essential to prioritize the optimization of air damping and thermoelastic damping, particularly in small gaps.

## Figures and Tables

**Table 1:** Structural parameters of CDRG.

Parameter	Value
Outermost ring diameter	3.8 mm
Center anchor diameter	1.7 mm
Width	13 μm
Spoke width	13.5 μm
Thickness	100 μm
Capacitive gap	7.5 μm
Resonant frequency	18,818 Hz

**Table 2:** Comparison of the results between the two models of MEMS (*T* = 298.15 K, *p* = 0.07 Pa).

Model	Continuous Fluid Model	Energy Transfer Model	Error Coefficient *k*
Squeeze film damping quality factor *Q_sq_*	2.075 × 10^7^	3.058 × 10^5^	66.86

**Table 3:** The typical properties of monocrystalline silicon materials under the (111) crystal orientation at room temperature (*T* = 298.15 K).

Material Attributes	Value	Unit
Density *ρ*	2330	Kg/m^3^
Young’s modulus *E*	168.9	G Pa
Poisson’s ratio *v*_//_ (horizontal direction)	0.262	-
Poisson’s ratio $v_{⊥}$ (vertical direction)	0.182	-
Modulus of shearing *G*_//_ (horizontal direction)	66.9	G Pa
Modulus of shearing $G_{⊥}$(vertical direction)	57.8	G Pa
Coefficient of expansion due to heat *α*	2.6 × 10^−6^	1/K
Heat conductivity *k*	130	W/(M·k)
Normal pressure heat capacity *C_v_*	713	J/(kg·K)

**Table 4:** Comparison of the *Q_TED_* between the analytical Zener model and the COMSOL simulation model for the CDRG.

Model	Zener Analytic Model	COMSOL Simulation Model	Error Coefficient
*Q_TED_*	2.452 × 10^5^	1.733 × 10^5^	41.5%

**Table 5:** System parameters of the CDRG.

Parameter	Value	Unit
Effective mass *m_eff_*	2.68 × 10^−7^	Kg
Resonant frequency *f*	18,818	Hz
Change rate (*dC/dx*)	2 × 10^−7^	F/m
Biased DC voltage *V_B_*	1.65	V
Equivalent input impedance@18,818 Hz *Z_in_*	1.88	MΩ
Feedback resistance *R_f_*	100	MΩ
Feedback capacitance *C_f_*	2	pF
Amplification gain of the amplifier	113	dB

**Table 6:** Theoretical calculation results of MEMS at room temperature (*T* = 298.15 K).

Damage Loss Mechanism	*Q*	Contribution Rate	*Q*	Contribution Rate
Air damping @ (0.07 pa)	2.075 × 10^7^ (Continuous fluid model)	~0.83%	3.058 × 10^5^ (Energy transfer model)	~36.14%
Thermoelastic damping	1.733 × 10^5^	~99.07%	1.733 × 10^5^	~63.79%
Anchor loss	1.921 × 10^8^	~0.089%	1.921× 10^8^	~0.058%
Other losses	1 × 10^9^	~0.017%	1 × 10^9^	~0.011%
Total loss	1.716 × 10^5^	-	1.105 × 10^5^	-

**Table 7:** Quality factor test results of CDRG prototypes (*T* = 298.15 K).

No.	$Q_{x}$	$Q_{y}$	ΔQ
#1	1.124 × 10^5^	1.13 × 10^5^	0.6 × 10^3^
#2	0.977 × 10^5^	0.974 × 10^5^	0.3 × 10^3^
#3	0.861 × 10^5^	0.885 × 10^5^	2.4 × 10^3^
#4	1.007 × 10^5^	1.025 × 10^5^	1.8 × 10^3^
#5	0.678 × 10^5^	0.659 × 10^5^	1.9× 10^3^
#6	1.23 × 10^5^	1.219 × 10^5^	1.1 × 10^3^
#7	0.957 × 10^5^	0.932 × 10^5^	2.5 × 10^3^
#8	1.108 × 10^5^	1.115 × 10^5^	0.7 × 10^3^

## Data Availability

The original contributions presented in the study are included in the article, further inquiries can be directed to the corresponding authors.
